# Biosynthetic Pathways of Ergot Alkaloids

**DOI:** 10.3390/toxins6123281

**Published:** 2014-12-10

**Authors:** Nina Gerhards, Lisa Neubauer, Paul Tudzynski, Shu-Ming Li

**Affiliations:** 1 Philipps-Universität Marburg, Institut für Pharmazeutische Biologie und Biotechnologie, Deutschhausstrasse 17A, D-35037 Marburg, Germany; E-Mail: nina.gerhards@pharmazie.uni-marburg.de; 2 Institut für Biologie und Biotechnologie der Pflanzen, Westfälische Wilhelms Universität Münster, Schlossplatz 8, D-48143 Münster, Germany; E-Mails: l_neub04@uni-muenster.de (L.N.); tudzyns@uni-muenster.de (P.T.)

**Keywords:** ergot alkaloids, biosynthetic pathway, secondary metabolism, natural products, fungi, mycotoxins

## Abstract

Ergot alkaloids are nitrogen-containing natural products belonging to indole alkaloids. The best known producers are fungi of the phylum Ascomycota, e.g., *Claviceps*, *Epichloë*, *Penicillium* and *Aspergillus* species. According to their structures, ergot alkaloids can be divided into three groups: clavines, lysergic acid amides and peptides (ergopeptines). All of them share the first biosynthetic steps, which lead to the formation of the tetracyclic ergoline ring system (except the simplest, tricyclic compound: chanoclavine). Different modifications on the ergoline ring by specific enzymes result in an abundance of bioactive natural products, which are used as pharmaceutical drugs or precursors thereof. From the 1950s through to recent years, most of the biosynthetic pathways have been elucidated. Gene clusters from several ergot alkaloid producers have been identified by genome mining and the functions of many of those genes have been demonstrated by knock-out experiments or biochemical investigations of the overproduced enzymes.

## 1. Introduction

Ergot alkaloids were named for the first known producer, the ergot fungus *Claviceps purpurea* (*C. purpurea*). This fungus is able to infect rye and other grains, and has caused several epidemics, particularly during the middle ages, due to consumption of rye products contaminated with *C. purpurea* sclerotia (ergots) [1,2]. The resulting disease is called ergotism or St. Anthony’s fire [1,2,3]. Patients show various symptoms depending on the amount and kind of alkaloids they consume. Painful spasms, diarrhea, paresthesia, nausea and vomiting, headache or psychosis are typical convulsive symptoms and gangrenous symptoms are observed especially for fingers and toes [1,2,3,4].

Ergot alkaloids show strong interactions with serotonin, dopamine and adrenergic receptors of the central nervous system and also with adrenergic receptors in blood vessels. Therefore, they can act as potent drugs. Examples with pharmaceutical applications are methylergometrine used in gynecology to stop bleeding after childbirth, ergotamine used to treat migraines, and the semi-synthetic derivative bromocriptine used to treat Parkinson’s disease [1,2,3,4]. The pharmacological activities can be explained by the structural similarity of ergot alkaloids with the three neurotransmitters.

**Figure 1:**
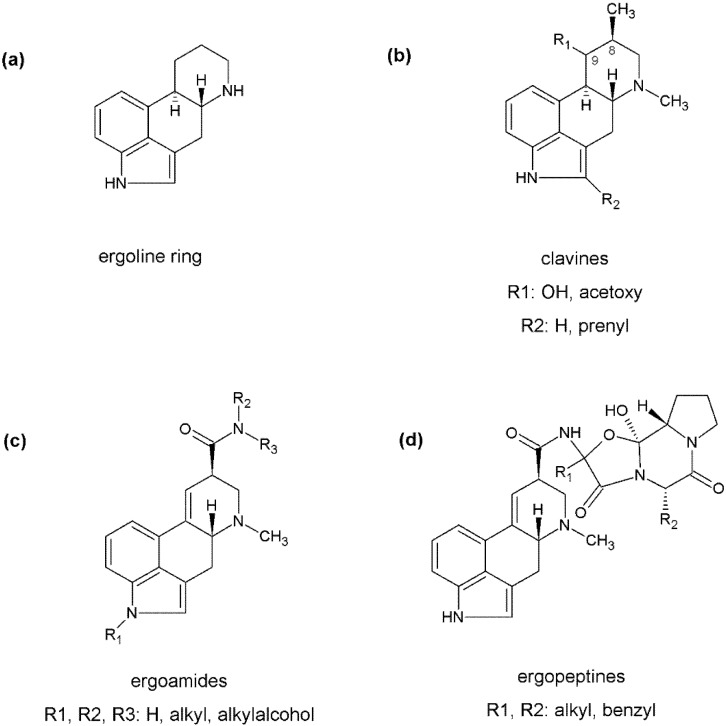
Chemical structures of ergot alkaloids: (**a**) ergoline ring (core structure of all ergot alkaloids); (**b**) core structure of clavines; (**c**) core structure of ergoamides; and (**d**) core structure of ergopeptines.

Ergot alkaloids with their common structure, the tetracyclic ergoline ring (Figure 1a) [5], can be divided into three groups: clavines, ergoamides, ergopeptides (ergopeptines). An exception is the simple clavine, chanoclavine, which lacks a cyclized d-ring. The natural clavines are sometimes modified on the ergoline scaffold with hydroxyl, acetoxyl or prenyl groups (Figure 1b) [4]. In contrast, natural lysergic acid derivatives are generally modified by a *C*9-amide linkage to give either the simple ergoamides (Figure 1c), or the complex ergopeptines that contain a cyclic tripeptide cyclol structure at this position (Figure 1d).

Ergot alkaloids are mainly produced by different fungi belonging to the phylum Ascomycota [4,6]. The fungal genera so far known to contain ergot alkaloid gene clusters are *Claviceps* [7,8], *Epichloë* (including *Neotyphodium* spp.), *Atkinsonella*, *Balansia*, *Periglandula* [9,10] and *Metarhizium* [9] in the family Clavicipitaceae, *Aspergillus* [11] and *Penicillium* [12] in the family Aspergillaceae, and *Arthroderma* (*Trichophyton*) [13] in the family Arthrodermataceae. Furthermore, ergot alkaloids can be found in species of the plant families of Convolvulaceae [14,15,16] and Poaceae [17] that are hosts for fungi in the Clavicipitaceae [18], and ergot alkaloids also reported from *Securidaca longipedunculata* in the plant family Polygalaceae [19].

## 2. Identification and Comparison of Biosynthetic Gene Clusters

Feeding experiments with precursors of the ergoline ring have shown that the skeleton is derived from l-tryptophan and dimethylallyl diphosphate (DMAPP) [20,21,22]. The first enzyme in the biosynthesis of ergot alkaloids, which catalyzes the *C*4-prenylation of l-tryptophan, was purified from a *Claviceps* sp. by Gebler and Poulter in 1992 [23]. Later the gene *dmaW*, encoding that enzyme, was isolated and sequenced, and the prenyltransfer reaction was demonstrated in recombinant yeast cells [24].

Tudzynksi *et al.*, identified a gene cluster containing the *dmaW* gene in *C. purpurea* strain P1 by genomic walking [25]. This cluster contains 14 genes that are involved in the biosynthesis of the ergopeptines ergotamine and ergocryptine [26,27,28], and the ergoamide, ergonovine. Since the mid-2000s, several fungal genomes have been sequenced and the biosynthetic genes for ergot alkaloids have been identified by genome mining and comparison with those of *C. purpurea* [4]. Nine homologous genes for the biosynthesis of ergot alkaloids have been identified in *Claviceps fusiformis*, which lacks functional copies of the nonribosomal peptide synthethase (NRPS) genes, in keeping with the absence of ergoamides and ergopeptines in cultures of this fungus [8]. In strains of the symbiotic endophyte *Epichloë festucae*, including var. *lolii* (*Neotyphodium lolii* from ryegrass [29], 12 homologues have been found in a cluster that determines biosynthesis of ergovaline [30]. In *Aspergillus fumigatus* (*A. fumigatus*), the end product of the ergot alkaloid biosynthesis is fumigaclavine C, and the cluster contains seven homologous genes plus additional genes likely to encode the modifying enzymes [20,31]. From a cosmid library of *Penicillium commune* (*P. commune*) Unsöld *et al.*, have identified a gene cluster with seven homologues and additional genes for the production of fumigaclavine A [32]. The corresponding gene clusters in species of the Arthrodermataceae include only five genes homologous to those of the *C. purpurea* gene cluster, indicating that a different pathway end product arises from that in *Aspergillus* or *Claviceps* spp. [13]. These five genes are found in all of the ergot alkaloid-producing fungi and are responsible for the formation of chanoclavine-I aldehyde (see below). The phylogenetic relationships of the biosynthetic genes and gene clusters of ergot alkaloids in different strains will be discussed elsewhere in this issue in a paper contributed by Gerhards *et al*.

## 3. Formation of the Ergoline Ring—Common Steps

The formation of the ergoline scaffold in the fungi *A. fumigatus* and *C. purpurea* has been investigated over the past seven decades. The functions of seven genes for the biosynthesis of the tetracyclic ergoline ring system have been demonstrated by knock-out-experiments and biochemical investigations [4].

**Figure 2:**
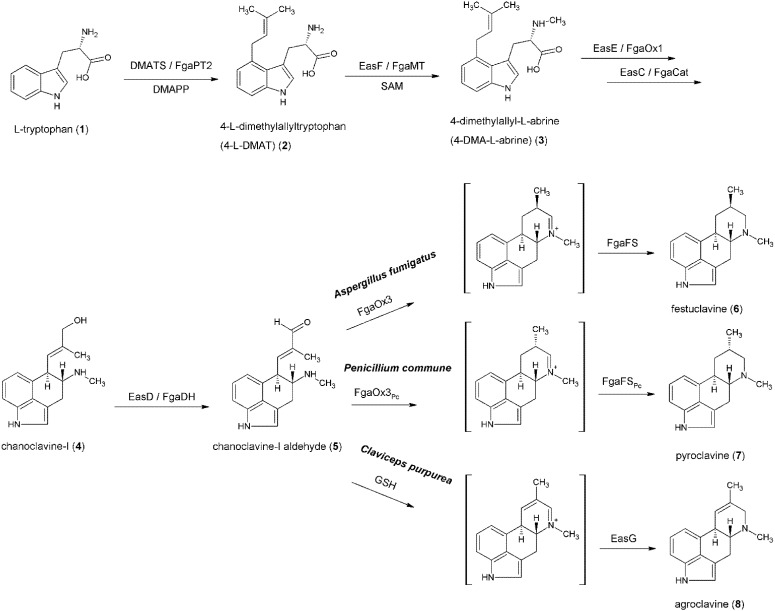
Formation of the ergoline scaffold-biosynthetic pathway.

The biosynthetic pathway is shown in Figure 2, which starts with the *C*4-prenylation of l-tryptophan (**1**) with dimethylallyl diphosphate (DMAPP) as prenyl donor. This reaction is catalyzed by the prenyltransferase 4-dimethylallyltryptophan synthase (DMATS), also named FgaPT2 in *A. fumigatus* [33,34,35]. Biochemical and structural elucidations clearly show the formation of 4-γ,γ-dimethylallyltryptophan (DMAT (**2**)) as product [36,37,38]. Metzger *et al.* reported the X-ray structure of FgaPT2 in complex with l-tryptophan, which led to a better understanding of the reaction mechanism [38,39]. They proposed a three-step mechanism: the formation of a dimethylallyl cation, a nucleophilic attack of the indole nucleus to that cation and a deprotonation step. Evolutionary investigations have indicated that the gene *fgaPT2* from *A. fumigatus* has the same origin as prenyltransferase genes from other Ascomycota, including the ergot-alkaloid-producing Clavicipitaceae [40].

Identification of the prenyltransferase, DMATS, has opened a new research field on enzymes of this superfamily, members of which catalyze the transfer of a prenyl moiety onto various aromatic substances, using DMAPP as the prenyl donor [41]. Recently, Liebhold *et al.*, showed the acceptance of unnatural alkyl donors by different indole prenyltransferases including FgaPT2 [42,43]. These studies have demonstrated the versatility of these enzymes towards alkyl donors and acceptors, which can catalyze *C*-, *O*- or *N*-prenylations of various aromatic compounds in different orientations (regular or reverse). This feature broadens the utility of these prenyltransferases for chemoenzymatic synthesis of prenylated compounds [41,44].

The second pathway-specific enzyme that has been characterized biochemically is the 4-dimethylallyltryptophan *N*-methyltransferase EasF (also named FgaMT in *A. fumigatus*). It catalyzes the *N*-methylation of **2** in the presence of S-adenosylmethionine (SAM), resulting in the formation of 4-dimethylallyl-l-abrine (4-DMA-l-abrine (**3**)). Rigbers and Li have demonstrated the formation of **3** with FgaMT from *A. fumigatus in vitro*. According to their report, this enzyme also shows broad substrate specificity [45].

The next intermediate in the pathway is chanoclavine-I (**4**). Gröger and Floss suggested that the conversion from **3** to **4** requires at least one decarboxylation and two oxidation steps [5,18]. There is evidence that at least two enzymes are needed for this conversion: the FAD-dependent oxidoreductase, EasE, and the catalase EasC (known as FgaOx1 and FgaCat in *A. fumigatus*, respectively) [46,47]. Lorenz *et al.*, have deleted the *easE* gene in *C. purpurea* strain P1 resulting in an accumulation of **3** and traces of **2**, whereas other intermediates of the pathway could not be detected, indicating the essential role of EasE for the formation of **4** [46]. Complementation of the mutant with functional *easE* restored ergot alkaloid biosynthesis. Furthermore, Goetz *et al.* performed a similar experiment with the *easC* gene. They disrupted the gene in *A. fumigatus*, which caused a block in the ergot alkaloid production beyond 4-DMA-l-abrine [47]. In addition, they were able to reproduce the results reported by Lorenz *et al*.

**Figure 3:**
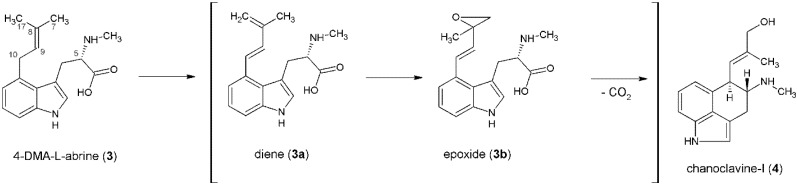
Postulated reaction mechanism for the formation of chanoclavine-I from 4-DMA-l-abrine via a diene and an epoxide intermediate.

Complementation of the *easC* or *easE* mutants with the respective wild type gene, or feeding chanoclavine-I to the mutants restored ergot alkaloid production. They concluded that both enzymes are required for the formation of **4**. However, this reaction is not yet understood in detail because all attempts to express and purify FgaOx1 have failed and purified EasC on its own has not been observed to catalyze the reaction under *in vitro* conditions [47]. Figure 3 shows the postulated reaction mechanism for the conversion of **3** to **4** (modified after Lorenz *et al.* [46]). A diene intermediate (**3a**) is formed by desaturation of the C8-C9 bond (possibly via an unstable intermediate that is hydroxylated at the benzyl carbon) and deprotonation at C17 [48]. The second oxidation takes place at the C7–C8 bond, which is followed by a decarboxylation of the epoxide intermediate (**3b**) [5,18,46]. Recent studies by Ryan *et al.* and Nielson *et al.*, support the hypothesis of the involvement of EasE and EasC in the formation of **4** [49,50]. Ryan *et al.*, did a partial reconstruction of the alkaloid pathway by amplifying a fragment of genomic DNA containing *fgaPT2*, *fgaMT*, *fgaOx1* and *fgaCat* from *A. fumigatus* and transformed it into *Aspergillus nidulans*. The strain that contained all four genes produced chanoclcavine-I. In contrast, other strains either with *fgaPT2*, *fgaMT* and *fgaOx1* or with *fgaPT2*, *fgaMT* and *fgaCat* showed an accumulation of 4-DMA-l-abrine [49]. The authors mentioned that they could not exclude the possibility that *A. nidulans* naturally harbors an enzyme that is also able to catalyze the formation of **4** from **3**, because of the high degree of relationship between the two fungi. The work of Nielsen *et al.*, excluded this possibility by using *Saccharomyces cerevisiae* as a host strain for the production of chanoclavine-I with four homologous genes from *Aspergillus japonicus* [50]. They confirmed the results of Ryan *et al.* [49], further indicating the involvement of EasE and EasC in the formation of **4**. Nielsen *et al.* [50], in addition, described the detection of a signal peptide sequence at the *C*-terminus of EasE. They proposed that this enzyme needs to pass through the secretory pathway for correct folding and formation of disulfide bonds before it can join up with EasC [50]. It remains unclear, if EasC performs a catalytic action in this reaction or if it has only the task to disproportionate H_2_O_2_ that is produced by EasE or other oxidases during the ergot alkaloid pathway [47,50].

The next intermediate in the pathway is chanoclavine-I aldehyde (**5**). Its formation is catalyzed by the short-chain dehydrogenase/reductase (SDR) EasD (named FgaDH in *A. fumigatus*). Wallwey *et al.* [51], have shown the formation of **5** from **4** by purified FgaDH in the presence of NAD^+^. FgaDH shows no significant sequence similarities to known SDRs and therefore represents a new group of short-chain dehydrogenases [51].

After the aforementioned reaction, the pathway reaches a branch point. A number of products arise from **5**, depending on the fungus. For example, the next intermediate in *A. fumigatus* is festuclavine (**6**), in *P. commune* pyroclavine (**7**) and in *C. purpurea* agroclavine (**8**) [52]. The branch point is mainly controlled by the old yellow enzyme EasA (also termed FgaOx3). Disruption of the *fgaOx3* gene in *A. fumigatus* leads to the accumulation of **4** and **5** [53], and the biosynthesis of the downstream ergot alkaloids can be restored by complementation with the wild-type gene. When *easA* from *C. purpurea* was used to complement the deleted gene from an *A. fumigatus* mutant, agroclavine accumulated. This indicates that functional differences in those enzymes result in divergent ergot alkaloid pathways [53]. For the formation of festuclavine in *A. fumigatus*, a second enzyme (the festuclavine synthase FgaFS) is required, as shown by Wallwey *et al.* [54,55]. They incubated both enzymes simultaneously or in tandem, together with **5** and the cofactors FMN and NADH, and demonstrated that festuclavine was the main product. Cheng *et al.*, reported the formation of agroclavine catalyzed by an enzyme from *E. festucae* var. *lolii* [56]. However, in *C. purpurea in vitro* investigations on the respective reaction showed that EasG (a homologue of FgaFS from *A. fumigatus*) is able to catalyze the formation of **8** via a non-enzymatic adduct with reduced glutathione [57]. As shown by Matuschek *et al.*, the formation of pyroclavine in *P. commune* requires both homologues: FgaOx3_PC_ and FgaFS_PC_ [52].

## 4. Formation of Fumigaclavines in *Aspergillus fumigatus* and *Penicillium commune*

**Figure 4:**
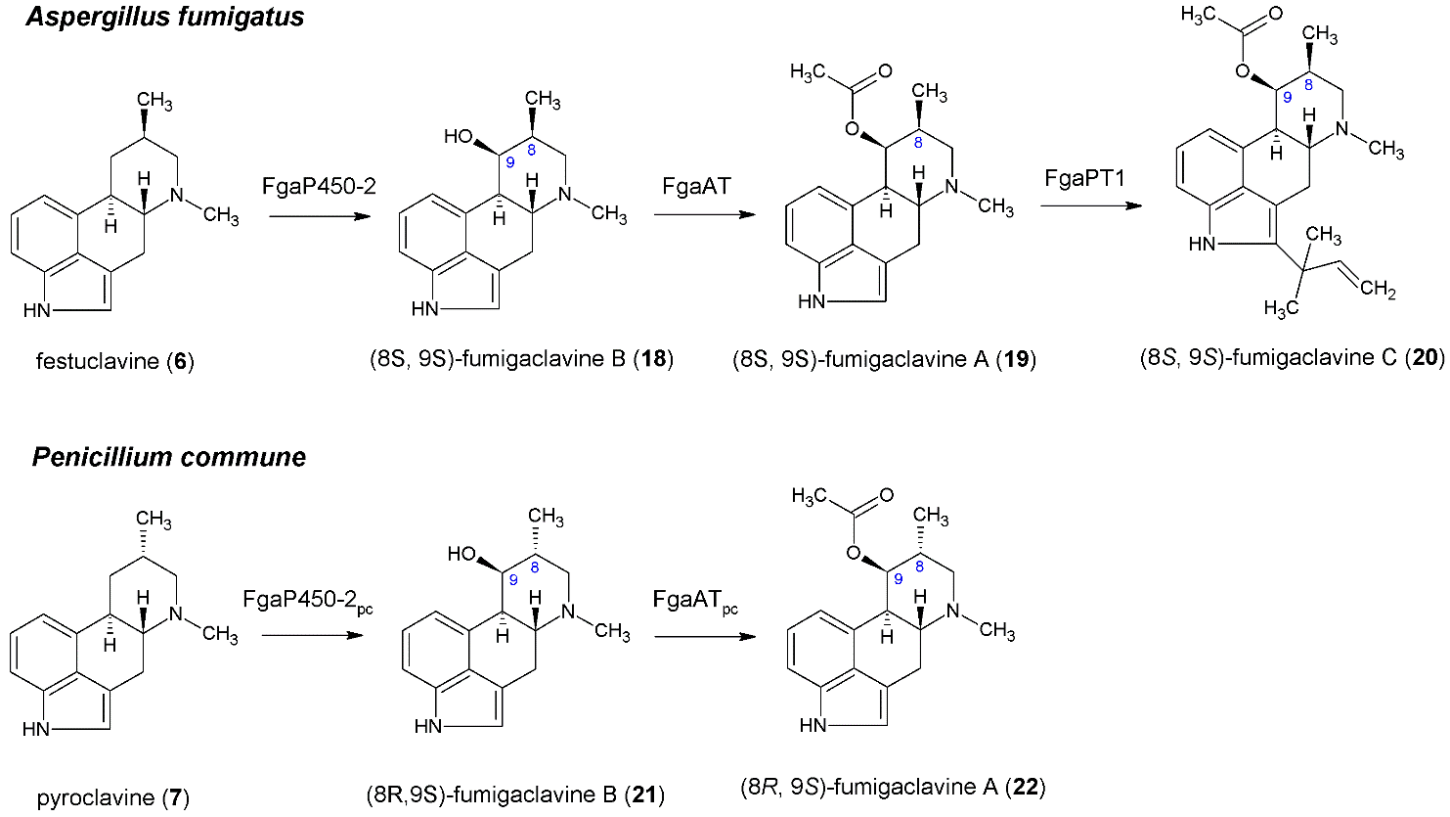
Formation of fumigaclavines in *Aspergillus fumigatus* and *Penicillium commune*.

Li and Unsöld analyzed the ergot alkaloid gene clusters of *A. fumigatus* and *C. purpurea* and showed that the cluster of *A. fumigatus* contains no peptide synthetase genes. Instead, genes coding for a putative hydroxylase (FgaP450-2), an *O*-acyltransferase (FgaAT) and another prenyltransferase (FgaPT1) are located in the cluster, which are absent in that of *C. purpurea* [20]. The postulated pathway for fumigaclavine C is shown in Figure 4. Isomers of fumigaclavine B (**18**, **21**) are formed from **6** and **7** via a hydroxylation in *A. fumigatus* and *P. commune*, respectively. This reaction is probably catalyzed by the monooxygenase FgaP450-2 in *A. fumigatus* and its orthologue FgaP450_PC_ in *P. commune* [4]. Biochemical evidence for this hypothesis is still outstanding. Liu *et al.*, investigated the conversion of **18** to fumigaclavine A (**19**), which is catalyzed by the acetyltransferase FgaAT in the presence of acetyl-CoA [58]. Three years before, the function of the prenyltransferase FgaPT1 was proven, which is responsible for the prenylation of **19** to yield fumigaclavine C (**20**), the end product of this pathway. This reaction requires DMAPP as prenyl donor, as shown before for the prenylation of **1** by FgaPT2 [59]. In *P. commune*, fumigaclavine A (**22**) was suggested to be the end product of the pathway, because the gene cluster lacks a second prenyltransferase gene [32].

Although fumigaclavine C contains no peptide moiety in its structure and the associated gene cluster in *A. fumigatus* lacks NRPS genes, O’Hanlon *et al.*, have reported the essential role of two NRPS genes *pesL* and *pes1* for the formation of fumigaclavine C [60]. Knock-out experiments have shown a complete loss of fumigaclavine C production and a corresponding increase in production of fumitremorgins after deletion of either *pesL* or *pes1*. The fact that all other ergot alkaloids including fumigaclavine A can still be found in extracts of the deletion mutants indicates the importance of the two enzymes in the final step of the pathway.

## 5. Formation of Lysergic Acid in *Claviceps purpurea*

**Figure 5:**
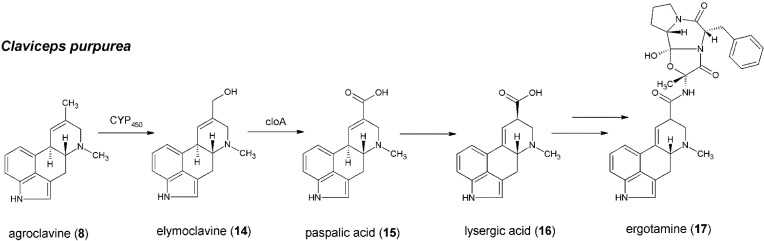
Formation of lysergic acid and ergotamine from agroclavine in *C. purpurea*.

The biosynthesis of lysergic acid from agroclavine remains largely unelucidated. Though multiple steps have been postulated to involve cytochrome P-450 monooxygenases [61], only one such enzyme is encoded in the ergot alkaloid biosynthesis cluster [12]. Elymoclavine, paspalic acid and lysergic acid have been identified as intermediates in the biosynthesis of lysergic acid [21] (Figure 5).

Elymoclavine (**14**) is formed from agroclavine (**8**) via a 2-electron oxidation and is further converted to paspalic acid (**15**) via a 4-electron oxidation [5]. These reactions have all been proposed to be catalyzed by cytochrome P-450 monooxygenases [61]. Kim *et al.*, showed the conversion of **14** to **15** by a microsomal fraction of *Claviceps* sp. in the presence of NADPH [62]. Cytochrome P-450 inhibitors blocked the conversion, suggesting that the responsible enzymes are cytochromes P-450. Disruption of a gene coding for cytochrome P-450 in *C. purpurea* [63] abolished the formation of ergopeptines and led to an accumulation of **4**, **8** and **14**. Feeding the mutant with d-lysergic acid restored the ergopeptine biosynthesis. The authors concluded that the mutant was blocked in the conversion of **14** to **16**, and named the gene *cloA* (for a clavine oxidase) [63]. Lorenz *et al.* [8] further investigated the formation of lysergic acid in *C. purpurea*, comparing its enzymes with those of *C. fusiformis*, which is only able to produce **8** and **14**, but no **16** or ergopeptines. Expression of *cloA* from *C. purpurea* in *C. fusiformis* resulted in the production of **16**. In contrast, expression of *cloA* from *C. fusiformis* in a *C. purpurea cloA* mutant did not complement the mutant to give these products. These results suggest that *C. fusiformis cloA*, though expressed and not obviously defective, is inactive in conversion of elymoclavine to paspalic acid. Schardl *et al.*, discuss the possibility that *cloA* can catalyze the formation of **14** as well as the formation of **15**, although they described some indications for the involvement of another enzyme [5]. Very recently, Robinson *et al.*, have co-expressed *easH*, *cloA* and *easA* from *Epichloë festucae* var. *lolii* × *Epichloë typhina* in a mutant strain of *A. fumigatus*, which typically does not produce ergot alkaloids derived from agroclavine [64]. Their data confirmed that CloA is able to catalyze multiple reactions, and conversion of **8** to **16** was detected in strains expressing *easA* and *cloA*. In the absence of *cloA*, the mutants expressing *easA* accumulated **8** and converted it into two novel ergot alkaloids [64].

Finally, conversion of **15** to **16** can be achieved either by catalysis of an isomerase or spontaneously, as already observed *in vitro* [5,21]. d-lysergic acid is an important link between the clavine pathway and the formation of ergopeptines such as ergotamine (**17**) and ergoamides, as described in Section 6.

## 6. From Lysergic Acid to Ergoamides and Ergopeptines

**Figure 6:**
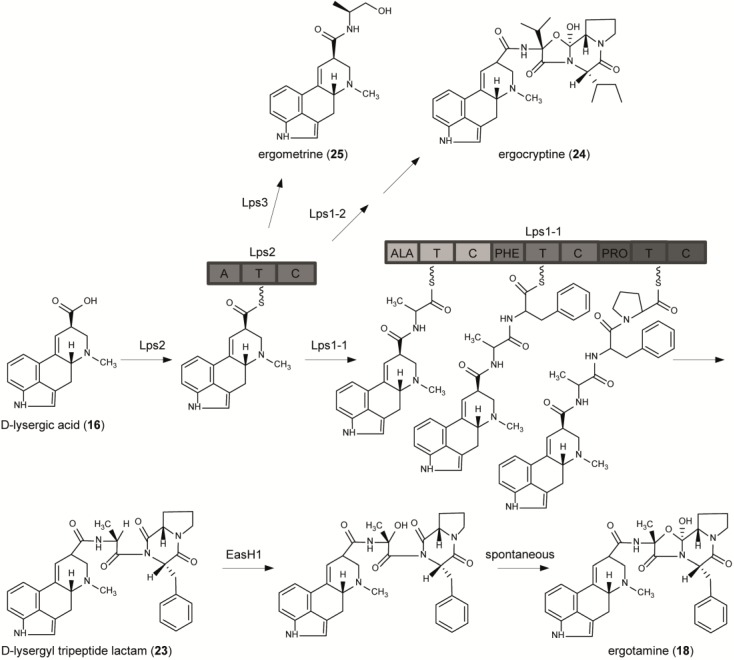
Formation of ergonovine and ergopeptines in *C. purpurea*.

The terminal pathway in *C. purpurea*, *Balansia obtecta*, and *Epichloë* species leading to ergopeptines includes the attachment of a tripeptide chain to activated lysergic acid, the tripeptide forming a bicyclic structure including a lactam ring and an oxazolidinone ring (e.g., ergotamine **18**). The different steps of this pathway have been elucidated in *C. purpurea* by classical *in vitro* enzymatic studies, gene knock-out approaches and enzymatic studies using protein preparations from *E. coli* transformants expressing the respective genes. Keller and co-workers showed that the d-lysergyl tripeptide lactams (**23**), the precursors of ergopeptines, are formed by an NRPS enzyme complex containing two separable activities, d-Lysergyl peptide synthetases 1 and 2 (LPS1 and LPS2) [65,66]. LPS2 catalyzes the first step, the generation of activated lysergic acid (hence it was predicted to be a monomodular NRPS), which is subsequently transferred to the large (trimodular) LPS1, where the d-lysergyl mono-, di- and tripeptide thioester intermediates are formed, and finally the d-lysergyl tripeptide lactam is released (Figure 6). This concept of two interacting NRPS subunits, which is so far unique in fungi, was confirmed by a joint enzymatic/genetic approach by the groups of Keller and Tudzynski, as described below.

**Figure 7:**
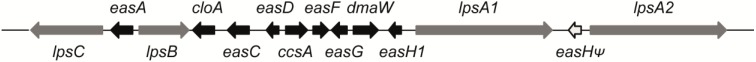
Ergot alkaloid gene cluster in *C. purpurea* (modified after [26]).

The EAS cluster in *C. purpurea* (Figure 7) contains four genes encoding non-ribosomal-peptide-synthetases (NRPS), two with three amino acid-activating modules each (*lpsA_1_*/*A_2_*), and two with a single module (*lpsB* and *C*) [25,26]. Functional analysis showed that *lpsB* encodes the d-lysergic acid activating enzyme LPS2 (Figure 6); this enzyme is quite unique because of the complex structure and hence bulkiness of its substrate [67].

The genes *lpsA*1 and *lpsA*2 both encode LPS1 enzymes, with LPS1-1 in strain P1 being involved in the synthesis of the major alkaloid ergotamine (including alanine, phenylalanine and proline), and LPS1-2 for the synthesis of ergocryptine (**24**) (valine, isoleucine, proline) [28]. Deletion of a comparable NRPS gene in a related endophyte, a hybrid *Epichloë* species symbiotic with perennial ryegrass, demonstrated its function in the biosynthesis of ergovaline [30]. The monomodular NRPS enzyme encoded by *lpsC* (ergonovine synthetase) catalyzes the formation of the d-lysergic acid alkanolamide, ergonovine (also called ergometrine or ergobasine) (**25**) by catalyzing condensation of alanine with lysergic acid, followed by reduction [68]. Recently Keller and co-workers elegantly showed that the small open reading frame of *easH*1 (see Figure 7) encodes an Fe^2+^/2-ketoglutarate-dependent dioxygenase that catalyzes the conversion of d-lysergyl tripeptide lactams to ergopeptines [69] (Figure 6). Thus, the NRPS complex encoded by genes of the *EAS* cluster in *C. purpurea* represents a unique natural combinatorial system whereby d-lysergic acid is activated by LPS2 and then used as substrate for any of the three different NRPSs, including LPS1 isoforms encoded by *lpsA*1 and *lps*A2, and the monomodular NRPS-reductase encoded by *lpsC*. Variations in LPS1 give many known ergopeptines, most of which vary in the first and second amino acid positions, but have proline in the third. The exception is ergobalansine, produced by *Balansia obtecta*, *Periglandula ipomoeae* and *Periglandula turbinae*, where the third position has l-alanine. This flexible biosynthesis scheme and the natural variability of the amino acid-binding domains of the LPS1 enzymes are the basis for the high variability of the ergot peptide alkaloid spectrum in the different natural chemical races of *C. purpurea*, as well as other Clavicipitaceae [70,71]. Knowledge of this system opens up interesting biotechnological perspectives to generate *C. purpurea* strains producing single alkaloids by knocking out *lpsA1/2* and/or *lpsC* genes (exemplified e.g., by Haarmann *et al.* and Correia *et al.* [28,67]), or even strains with new specificities by introducing “designer” *lps* genes.

## 7. Conclusions and Outlook

The biosynthetic pathway for ergot alkaloids has been investigated extensively in *Claviceps* species and *A. fumigatus*. Among the early steps, biochemical confirmation for the formation of chanoclavine-I, catalyzed by an oxidase and a catalase, remains to be accomplished in full. Among the later steps of the pathway, questions remain regarding the conversion of agroclavine to elymoclavine, and the specific LPS1 variations underlying ergopeptine diversity. Such questions could be addressed by heterologous expression and purification of the putative membrane-bound cytochrome P-450 monooxygenases and NRPS enzymes, though this is still a challenge.

The elucidation of the ergot alkaloid-biosynthesis pathway is of interest especially because of the broad range of pharmaceutical uses. With increased knowledge concerning the genes and enzymes, molecular genetic manipulations may be used to improve industrial production of medically important ergot alkaloids, and novel forms that could act as drugs with new or improved pharmacological activities and minimal side effects might be created by synthetic microbiology, semisynthetic synthesis or other related techniques.

## References

[1] Haarmann T., Rolke Y., Giesbert S., Tudzynski P. (2009). Ergot: From witchcraft to biotechnology. Mol. Plant Pathol..

[2] Schiff P.L. (2006). Ergot and its alkaloids. Am. J. Pharm. Educ..

[3] Jakubczyk D., Cheng J.Z., O’Connor S.E. (2014). Biosynthesis of the ergot alkaloids. Nat. Prod. Rep..

[4] Wallwey C., Li S.M. (2011). Ergot alkaloids: Structure diversity, biosynthetic gene clusters and functional proof of biosynthetic genes. Nat. Prod. Rep..

[5] Schardl C.L., Panaccione D.G., Tudzynski P. (2006). Ergot alkaloids—Biology and molecular biology. Alkaloids Chem. Biol..

[6] Boichenko L.V., Boichenko D.M., Vinokurova N.G., Reshetilova T.A., Arinbasarov M.U. (2001). Screening for ergot alkaloid producers among microscopic fungi by means of the polymerase chain reaction. Microbiology.

[7] Hulvova H., Galuszka P., Frebortova J., Frebort I. (2013). Parasitic fungus *Claviceps* as a source for biotechnological production of ergot alkaloids. Biotechnol. Adv..

[8] Lorenz N., Wilson E.V., Machado C., Schardl C.L., Tudzynski P. (2007). Comparison of ergot alkaloid biosynthesis gene clusters in *Claviceps* species indicates loss of late pathway steps in evolution of *C. fusiformis*. Appl. Environ. Microbiol..

[9] Gao Q., Jin K., Ying S.H., Zhang Y., Xiao G., Shang Y., Duan Z., Hu X., Xie X.Q., Zhou G. (2011). Genome sequencing and comparative transcriptomics of the model entomopathogenic fungi *Metarhizium anisopliae* and *M. acridum*. acridum. PLoS. Genet..

[10] Kozlovsky A.G., Zhelifonova V.P., Antipova T.V., Zelenkova N.F. (2011). Physiological and biochemical characteristics of the genus *Penicillium* fungi as producers of ergot alkaloids and quinocitrinins. Appl. Biochem. Microbiol..

[11] Ge H.M., Yu Z.G., Zhang J., Wu J.H., Tan R.X. (2009). Bioactive alkaloids from endophytic *Aspergillus fumigatus*. J. Nat. Prod..

[12] Kozlovsky A.G., Zhelifonova V.P., Antipova T.V. (2013). Fungi of the genus *Penicillium* as producers of physiologically active compounds. Appl. Biochem. Microbiol..

[13] Wallwey C., Heddergott C., Xie X., Brakhage A.A., Li S.M. (2012). Genome mining reveals the presence of a conserved gene cluster for the biosynthesis of ergot alkaloid precursors in the fungal family Arthrodermataceae. Microbiology.

[14] Beaulieu W.T., Panaccione D.G., Hazekamp C.S., Mckee M.C., Ryan K.L., Clay K. (2013). Differential allocation of seed-borne ergot alkaloids during early ontogeny of morning glories (Convolvulaceae). J. Chem. Ecol..

[15] Markert A., Steffan N., Ploss K., Hellwig S., Steiner U., Drewke C., Li S.M., Boland W., Leistner E. (2008). Biosynthesis and accumulation of ergoline alkaloids in a mutualistic association between *Ipomoea asarifolia* (Convolvulaceae) and a Clavicipitalean fungus. Plant Physiol..

[16] Ahimsa-Müller M.A., Markert A., Hellwig S., Knoop V., Steiner U., Drewke C., Leistner E. (2007). Clavicipitaceous fungi associated with ergoline alkaloid-containing Convolvulaceae. J. Nat. Prod..

[17] Schardl C.L., Young C.A., Pan J., Florea S., Takach J.E., Panaccione D.G., Farman M.L., Webb J.S., Jaromczyk J., Charlton N.D. (2013). Currencies of mutualisms: Sources of alkaloid genes in vertically transmitted *epichloae*. Toxins.

[18] Gröger D., Floss H.G. (1998). Biochemistry of ergot alkaloids—Achievements and challenges. Alkaloids Chem. Biol..

[19] Scandola M., Games D.E., Costa C., Allegri G., Bertazzo A., Curcuruto O., Traldi P. (1994). Structural study of alkaloids from *Securidaca longipedunculata* roots II. Isolation and characterization by supercritical fluid chromatography/mass spectrometry. J. Heterocycl. Chem..

[20] Li S.M., Unsöld I.A. (2006). Post genome research on the biosynthesis of ergot alkaloids. Planta Med..

[21] Floss H.G. (1976). Biosynthesis of ergot alkaloids and related compounds. Tetrahedron.

[22] Williams R.M., Stocking E.M., Sanz-Cervera J.F. (2000). Biosynthesis of prenylated alkaloids derived from tryptophan. Top. Curr. Chem..

[23] Gebler J.C., Poulter C.D. (1992). Purification and characterization of dimethylallyl tryptophan synthase from *Claviceps purpurea*. Arch. Biochem. Biophys..

[24] Tsai H.F., Wang H., Gebler J.C., Poulter C.D., Schardl C.L. (1995). The *Claviceps purpurea* gene encoding dimethylallyltryptophan synthase, the committed step for ergot alkaloid biosynthesis. Biochem. Biophys. Res. Commun..

[25] Tudzynski P., Holter K., Correia T., Arntz C., Grammel N., Keller U. (1999). Evidence for an ergot alkaloid gene cluster in *Claviceps purpurea*. Mol. Gen. Genet..

[26] Haarmann T., Machado C., Lübbe Y., Correia T., Schardl C.L., Panaccione D.G., Tudzynski P. (2005). The ergot alkaloid gene cluster in *Claviceps purpurea*: Extension of the cluster sequence and intra species evolution. Phytochemistry.

[27] Lorenz N., Haarmann T., Pazoutova S., Jung M., Tudzynski P. (2009). The ergot alkaloid gene cluster: Functional analyses and evolutionary aspects. Phytochemistry.

[28] Haarmann T., Lorenz N., Tudzynski P. (2008). Use of a nonhomologous end joining deficient strain (Deltaku70) of the ergot fungus *Claviceps purpurea* for identification of a nonribosomal peptide synthetase gene involved in ergotamine biosynthesis. Fungal Genet. Biol..

[29] Fleetwood D.J., Scott B., Lane G.A., Tanaka A., Johnson R.D. (2007). A complex ergovaline gene cluster in epichloe endophytes of grasses. Appl. Environ. Microbiol..

[30] Panaccione D.G., Johnson R.D., Wang J., Young C.A., Damrongkool P., Scott B., Schardl C.L. (2001). Elimination of ergovaline from a grass-*Neotyphodium* endophyte symbiosis by genetic modification of the endophyte. Proc. Natl. Acad. Sci. USA.

[31] Panaccione D.G., Coyle C.M. (2005). Abundant respirable ergot alkaloids from the common airborne fungus *Aspergillus fumigatus*. Appl. Environ. Microbiol..

[32] Unsöld I.A. (2006). Molecular Biological and Biochemical Investigations on the Biosynthesis of Fumigaclavines in *Aspergillus fumigatus* AF 293/B 5233 and *Penicillium commune* NRRL2033. Ph.D. Thesis.

[33] Lee S.L., Floss H.G., Heinstein P. (1976). Purification and properties of dimethylallylpyrophosphate: Tryptophan dimethylallyl transferase, the first enzyme of ergot alkaloid biosynthesis in *Claviceps*. sp. SD 58. Arch. Biochem. Biophys..

[34] Coyle C.M., Panaccione D.G. (2005). An ergot alkaloid biosynthesis gene and clustered hypothetical genes from *Aspergillus fumigatus*. Appl. Environ. Microbiol..

[35] Unsöld I.A., Li S.M. (2005). Overproduction, purification and characterization of FgaPT2, a dimethylallyltryptophan synthase from *Aspergillus fumigatus*. Microbiology.

[36] Steffan N., Unsöld I.A., Li S.M. (2007). Chemoenzymatic synthesis of prenylated indole derivatives by using a 4-dimethylallyltryptophan synthase from *Aspergillus fumigatus*. Chembiochem.

[37] Steffan N., Li S.M. (2009). Increasing structure diversity of prenylated diketopiperazine derivatives by using a 4-dimethylallyltryptophan synthase. Arch. Microbiol..

[38] Metzger U., Schall C., Zocher G., Unsöld I., Stec E., Li S.-M., Heide L., Stehle T. (2009). The structure of dimethylallyl tryptophan synthase reveals a common architecture of aromatic prenyltransferases in fungi and bacteria. Proc. Natl. Acad. Sci. USA.

[39] Luk L.Y.P., Tanner M.E. (2009). Mechanism of dimethylallyltryptophan synthase: Evidence for a dimethylallyl cation intermediate in an aromatic prenyltransferase reaction. J. Am. Chem. Soc..

[40] Liu M., Panaccione D.G., Schardl C.L. (2009). Phylogenetic analyses reveal monophyletic origin of the ergot alkaloid gene *dmaW* in fungi. Evol. Bioinform..

[41] Yu X., Li S.M. (2012). Prenyltransferases of the dimethylallyltryptophan synthase superfamily. Methods Enzymol..

[42] Liebhold M., Xie X., Li S.-M. (2012). Expansion of enzymatic Friedel-Crafts alkylation on indoles: Acceptance of unnatural beta-unsaturated allyl diphospates by dimethylallyl-tryptophan synthases. Org. Lett..

[43] Liebhold M., Li S.M. (2013). Regiospecific benzylation of tryptophan and derivatives catalyzed by a fungal dimethylallyl transferase. Org. Lett..

[44] Li S.M. (2009). Applications of dimethylallyltryptophan synthases and other indole prenyltransferases for structural modification of natural products. Appl. Microbiol. Biotechnol..

[45] Rigbers O., Li S.M. (2008). Ergot alkaloid biosynthesis in *Aspergillus fumigatus*: Overproduction and biochemical characterisation of a 4-dimethylallyltryptophan *N*-methyltransferase. J. Biol. Chem..

[46] Lorenz N., Olšovská J., Šulc M., Tudzynski P. (2010). The alkaloid cluster gene *ccsA* of the ergot fungus *Claviceps purpurea* encodes the chanoclavine-I-synthase, an FAD-containing oxidoreductase mediating the transformation of *N*-methyl-dimethylallyltryptophan to chanoclavine-I. Appl. Environ. Microbiol..

[47] Goetz K.E., Coyle C.M., Cheng J.Z., O’Connor S.E., Panaccione D.G. (2011). Ergot cluster-encoded catalase is required for synthesis of chanoclavine-I in *Aspergillus fumigatus*. Curr. Genet..

[48] Kozikowski A.P., Chen C., Wu J.P., Shibuya M., Kim C.G., Floss H.G. (1993). Probing ergot alkaloid biosynthesis: Intermediates in the formation of ring C. J. Am. Chem. Soc..

[49] Ryan K.L., Moore C.T., Panaccione D.G. (2013). Partial reconstruction of the ergot alkaloid pathway by heterologous gene expression in *Aspergillus nidulans*. Toxins.

[50] Nielsen C., Folly C., Hatsch A., Molt A., Schroder H., O’Connor S.E., Naesby M. (2014). The important ergot alkaloid intermediate chanoclavine-I produced in the yeast *Saccharomyces cerevisiae* by the combined action of EasC and EasE from *Aspergillus japonicus*. Microb. Cell Fact..

[51] Wallwey C., Matuschek M., Li S.M. (2010). Ergot alkaloid biosynthesis in *Aspergillus fumigatus*: Conversion of chanoclavine-I to chanoclavine-I aldehyde catalyzed by a short-chain alcohol dehydrogenase FgaDH. Arch. Microbiol..

[52] Matuschek M., Wallwey C., Wollinsky B., Xie X., Li S.M. (2012). *In vitro* conversion of chanoclavine-I aldehyde to the stereoisomers festuclavine and pyroclavine controlled by the second reduction step. RSC Adv..

[53] Coyle C.M., Cheng J.Z., O’Connor S.E., Panaccione D.G. (2010). An old yellow enzyme gene that controls the branch point between *Aspergillus fumigatus* and *Claviceps purpurea* ergot alkaloid pathways. Appl. Environ. Microbiol..

[54] Wallwey C., Matuschek M., Xie X.L., Li S.M. (2010). Ergot alkaloid biosynthesis in *Aspergillus fumigatus*: Conversion of chanoclavine-I aldehyde to festuclavine by the festuclavine synthase FgaFS in the presence of the old yellow enzyme FgaOx3. Org. Biomol. Chem..

[55] Xie X., Wallwey C., Matuschek M., Steinbach K., Li S.M. (2011). Formyl migration product of chanoclavine-I aldehyde in the presence of the old yellow enzyme FgaOx3 from *Aspergillus fumigatus*: A NMR structure elucidation. Magn Reson. Chem..

[56] Cheng J.Z., Coyle C.M., Panaccione D.G., O’Connor S.E. (2010). Controlling a structural branch point in ergot alkaloid biosynthesis. J. Am. Chem. Soc..

[57] Matuschek M., Wallwey C., Xie X., Li S.M. (2011). New insights into ergot alkaloid biosynthesis in *Claviceps purpurea*: An agroclavine synthase EasG catalyses, via a non-enzymatic adduct with reduced glutathione, the conversion of chanoclavine-I aldehyde to agroclavine. Org. Biomol. Chem..

[58] Liu X., Wang L., Steffan N., Yin W.B., Li S.M. (2009). Ergot alkaloid biosynthesis in *Aspergillus fumigatus*: FgaAT catalyses the acetylation of fumigaclavine B. ChemBioChem.

[59] Unsöld I.A., Li S.M. (2006). Reverse prenyltransferase in the biosynthesis of fumigaclavine C in *Aspergillus fumigatus*: Gene expression, purification and characterization of fumigaclavine C synthase FgaPT1. ChemBioChem.

[60] O’Hanlon K.A., Gallagher L., Schrettl M., Jochl C., Kavanagh K., Larsen T.O., Doyle S. (2012). Nonribosomal peptide synthetase genes *pesL* and *pes1* are essential for fumigaclavine C production in *Aspergillus fumigatus*. Appl. Environ. Microbiol..

[61] Maier W., Schumann B., Gröger D. (1988). Microsomal oxygenases involved in ergoline alkaloid biosynthesis of various *Claviceps* strains. J. Basic Microbiol..

[62] Kim S.U., Cho Y.J., Floss H.G., Anderson J.A. (1983). Conversion of elymoclavine to paspalic acid by a particulate fraction from an ergotamine-producing strain of *Claviceps* sp.. Planta Med..

[63] Haarmann T., Ortel I., Tudzynski P., Keller U. (2006). Identification of the cytochrome P450 monooxygenase that bridges the clavine and ergoline alkaloid pathways. ChemBioChem.

[64] Robinson S.L., Panaccione D.G. (2014). Heterologous expression of lysergic acid and novel ergot alkaloids in *Aspergillus fumigatus*. Appl. Environ. Microbiol..

[65] Riederer B., Han M., Keller U. (1996). d-Lysergyl peptide synthetase from the ergot fungus *Claviceps purpurea*. J. Biol. Chem..

[66] Walzel B., Riederer B., Keller U. (1997). Mechanism of alkaloid cyclopeptide synthesis in the ergot fungus *Claviceps purpurea*. Chem. Biol..

[67] Correia T., Grammel N., Ortel I., Keller U., Tudzynski P. (2003). Molecular cloning and analysis of the ergopeptine assembly system in the ergot fungus *Claviceps purpurea*. Chem. Biol..

[68] Ortel I., Keller U. (2009). Combinatorial assembly of simple and complex d-lysergic acid alkaloid peptide classes in the ergot fungus *Claviceps purpurea*. J. Biol. Chem..

[69] Havemann J., Vogel D., Loll B., Keller U. (2014). Cyclolization of d-lysergic acid alkaloid peptides. Chem. Biol..

[70] Keller U., Tudzynski P. (2002). Ergot alkaloids. Industrial Applications.

[71] Tudzynski P., Neubauer L. (2014). Ergot alkaloids. Biosynthesis and Molecular Genetics of Fungal Secondary Metabolites.

